# Quantifying the Modern City: Emerging Technologies and Big Data for Active Living Research

**DOI:** 10.3389/fpubh.2017.00105

**Published:** 2017-05-29

**Authors:** Deepti Adlakha

**Affiliations:** ^1^Center for Public Health, School of Medicine, Dentistry and Biomedical Sciences, Queen’s University Belfast, Belfast, UK; ^2^School of Natural and Built Environment, Queen’s University Belfast, Belfast, UK

**Keywords:** emerging technologies, big data, active living, built environment, physical activity, mobile applications, wearable technology

## Abstract

Opportunities and infrastructure for active living are an important aspect of a community’s design, livability, and health. Features of the built environment influence active living and population levels of physical activity, but objective study of the built environment influence on active living behaviors is challenging. The use of emerging technologies for active living research affords new and promising means to obtain objective data on physical activity behaviors and improve the precision and accuracy of measurements. This is significant for physical activity promotion because precise measurements can enable detailed examinations of where, when, and how physical activity behaviors actually occur, thus enabling more effective targeting of particular behavior settings and environments. The aim of this focused review is to provide an overview of trends in emerging technologies that can profoundly change our ability to understand environmental determinants of active living. It discusses novel technological approaches and big data applications to measure and track human behaviors that may have broad applications across the fields of urban planning, public health, and spatial epidemiology.

## Introduction

Emerging public health research has highlighted that environmental exposures are responsible for a majority of risk factors for many diseases (1–4). For example, adverse neighborhood conditions (poverty, racial segregation, unemployment, etc.) are known to affect various health outcomes and are independently associated with an increased risk of various diseases (4). Research has highlighted that neighborhood level characteristics may play an important role beyond characteristics at the individual level (3, 4). Neighborhood characteristics can be classified in multiple aspects, including the built/physical environment (e.g., availability of sidewalks, bicycle paths, parks, and open spaces) (5, 6), social environment (e.g., community cohesion) (7), economic conditions (e.g., poverty rate, unemployment), and environmental determinants (e.g., toxic pollutants, greenhouse gas emissions, and non-biodegradable waste) (8).

Researchers have urged for systematic and comprehensive measures of these exposures to quantify their impacts on human health. For example, most research on built and social environment exposures has used self-reported instruments or used census data (6, 9, 10). To date, studies seeking to quantify how features or changes in the built environment impact individuals’ health behaviors have employed extensive in-the-field observation, often providing a limited view of behaviors at a specific point in time, their context, and how each change as a function of the environment. What if, instead, we could conduct longitudinal observations of tens of thousands of environments over many years, track historical trends, and quantify how people react to changes in their environments over time? The advent of newly developing technologies such as webcams, social media, or crowdsourcing may offer new opportunities to obtain geo-spatial data about neighborhoods that may circumvent the limitations of traditional data sources used in neighborhood research. These new methods and technologies to measure exposures may provide a more balanced approach to the measurement of the gene environment equation (8).

A key population behavior of interest to our research team is active living and the promotion of activity-friendly neighborhoods and communities (11–13). Neighborhoods that are designed to be activity friendly can influence physical activity behaviors and promote active lifestyles by default, allowing residents to integrate physical activity into their daily routines. The purpose of this focused review is to provide an overview of trends in emerging technologies that can profoundly change our ability to understand environmental determinants of active living. It examines novel technological approaches and big data applications that are being used to enumerate global physical activity patterns and built environment characteristics with broad applications across the fields of urban planning, public health, and spatial epidemiology. This focused review builds on the findings of the original article by Adlakha et al. (12), discusses gaps in measurement, and highlights four key concepts in emerging technologies that may fill these gaps, thus providing new opportunities for active living research.

Physical activity is preventive factor for many chronic disease outcomes including obesity, diabetes, heart disease, and some cancers (14). The built environment can facilitate or constrain physical activity and is an important influence for promotion of active living (15). The design of built environments that promote physical activity and active mobility can play a key role in chronic disease prevention (16, 17). Lack of access to parks and open space and inadequate walking/bicycling infrastructure can impact the frequency, duration, and quality of physical activity available to residents in urban settings (2, 3, 6). Physical activity may be purposive such as a jog in a park, or incidental such as a 10 min walk from home to a public transit stop (17). In both purposive and incidental cases, the design of built environments influences the decisions and experience of these behaviors (6, 18). For example, multimodal public transit systems can encourage people to opt for transport options that integrate physical activity (e.g., walking, bicycling) as a part of daily life as opposed to motorized and carbon-dependent means. In the UK, for example, over 50% of car journeys are under 5 km (19, 20). Recent studies have shown that these automobile journeys can be easily replaced with more active forms of travel (21). This practice offers multiple benefits of increasing levels of physical activity and reducing rates of obesity, while reducing the consumption of fossil fuels and consequent pollution. Research has suggested that the evaluation of existing environments is crucial to the implementation of programs and interventions for active living within the built environment. Features of the built environment influence active living and travel decisions, but objective study of the effects of built environment improvements on active living remains challenging. Several obstacles remain to undertake evaluation of the built environment and human behaviors in an efficient and cost-effective manner.

In 2014, a team led by Adlakha et al. was the first of its kind to use publicly available web data feeds, along with Geographic information system (GIS) to assess the use of parks and green spaces for physical activity (bouts of running and walking) in a Midwestern US city (St. Louis, MO, USA) (12). Running and walking bouts downloaded from the route mapping website https://MapMyRun.com were used to identify differences in outdoor physical activity. A key strength of this study was the novel use of large-scale data from which provided a cheaper alternative for tracking of physical activity across larger spatial and temporal settings. This study was the first of its kind to use unobtrusive, inexpensive, and publicly available web data feeds. It demonstrated the potential of big data approaches and emerging technologies to advance physical activity tracking and measurement by placing minimum burden on the sample population.

Adlakha et al. (18) also highlighted that existing public health literature on physical activity and settings where it occurs (e.g., parks, green spaces, etc.) focuses on proximity to destinations and land-use, but little is known about the absolute amount or types of activity they facilitate and the sociodemographic characteristics of the populations they serve (12). The use of direct observation tools [e.g., System for Observing Physical Activity and Recreation in Communities (22), Block Walk Method (23)] have overcome these constraints in their ability to provide objective, contextually rich information on physical activity within settings, but these data are static since parks are divided into predetermined target areas and then studied by trained observers (22). Other limitations of direct observation instruments are the time-intensive nature and costs involved in data collection (24). In contrast, big data and emerging technologies can provide a cheaper, objective alternative for precise tracking of physical activity behaviors across larger spatial and temporal settings.

KEY CONCEPT 1. Big data and emerging technologies Big data and modern technology has opened up several opportunities to obtain new insights on cities and offer the potential for dramatically more efficient measurement tools (25, 26). Emerging technologies are technical innovations, which represent prominent and progressive ongoing developments and advances in various fields of modern technology for competitive advantage. They are characterized by radical novelty, relatively fast growth, coherence, and the potential to exert prominent impact in the future. The emergence phase of new technological innovations is often uncertain and ambiguous (27). Big data refers to, “an exceptionally large volume and variety of data that may be analyzed computationally to reveal patterns, trends, and associations, especially relating to human behavior and interactions” (28–30). Novel methods, such as those offered by big data or non-traditional sources may be viable tools for filling gaps in traditional data collection methods. For example, crowdsourcing, large imagery databases, and mobile apps are three types of big data that could profoundly change our ability to understand environmental determinants of physical activity and inform policies to implement changes in neighborhoods.

The application of technology in the fields of architecture, urban design, city and regional planning, as well as the disciplines of social sciences like psychology, sociology, and geography goes back in time. In the late 1960s and 1970s, the American sociologist and urban theorist William H. Whyte produced the Street Life Project and conducted revolutionary, cutting-edge granular studies of outdoor public spaces in the city of New York (31, 32). Whyte spent several hours filming, photographing, and taking notes about human behavior in public spaces. He explored the seemingly mundane, but important design questions of where people liked to sit, stand, gather, linger, meet friends, and engage in conversations. Whyte believed that if planners and designers knew how the placement of street furniture like benches and street lamps, a plaza’s orientation to the sun, or shade from trees affected people’s enjoyment of a public space, then the field of urban planning could go beyond mere observation into the realm of smarter policy and placemaking. Placemaking refers to a collaborative, community-based participatory process of shaping the public realm in order to maximize shared value (33, 34).

An effective placemaking process capitalizes on a local community’s assets, inspiration, and potential, and it results in the creation of quality public spaces that contribute to people’s health, happiness, and well-being. More than just promoting better urban design, placemaking facilitates creative patterns of use, paying particular attention to the physical, cultural, and social identities that define a place and support its ongoing evolution (34). The Street Life Project was ground-breaking for the field of urban planning and changed not only the way we think about public spaces but also what can be learned in this kind of close observational research of human behavior and interaction. Around the same time, Kevin Lynch’s published his seminal work titled, “The Image of the City,” which was one of the first to emphasize the importance of social scientists and design professionals in signifying ways that urban design and built environments can be quantitatively measured and improved (35). The work of Whyte and Lynch was ground breaking and has influenced enormous efforts to investigate the structure and function of cities, to characterize perception of neighborhoods (36, 37), and promotion of social interactions (38, 39).

## Emerging Trends for Evaluation of Built Environment and Human Behaviors

To date, large-scale evaluation studies have required extensive in-the-field observation using audits and/or data-intensive technologies including accelerometers and geographic positioning systems (GPS) (40, 41). Such studies only provide a limited view of behaviors, their context, and how each may change as a function of the built environment. Studies using environmental audits are often costly in time, personnel, and financial resources, deploying masses of graduate students to conduct interviews about people’s daily routines (38), or requiring hand-coding of thousands of hours of video (32) to characterize a few city plazas and parks. Simple walkability audits require a specifically trained observer to travel to street segments and assess the environment characteristics. Limitations of video recordings include the time and labor-consuming process to annotate video feeds and researcher bias introduced during the analysis process (42). As an alternative, Google Street View provides an efficient and reliable method to remotely conduct visual audits of neighborhoods and street spaces (42–45). GIS has also been used to map spatial data and analyze neighborhood characteristics (46, 47). Current state-of-the art technology to investigate associations between human behavior and the urban built environment has employed multiple expensive devices at the individual level (e.g., GPS, accelerometer) and connected this data to GIS layers (27, 48, 49).

The ubiquity of technology and technological advancements have led to the use of cameras, video, and other computer-based technologies in a number of fields to conduct surveillance of plazas, roadways, sidewalks, crosswalks, and other public locations. The relative ease of capturing large-scale data has led to opportunities to map urban environments with promising results that highlight how people move through cities based on check-ins (50, 51), uploaded photos (52), or live video feeds. Electronic audits of neighborhood built environment spaces gives researchers and urban planners a means to increase their evaluative capacity in less time, using fewer resources than in-person audits. In addition, GIS, GPS, accelerometers, smart phone mobile applications (apps), wearable technology that includes activity trackers and devices such as person-based point of view wearable cameras, webcams and imagery databases, are each being used in many studies, often in combination (26, 47, 48). These large volumes of data can improve our understanding of health behaviors by providing insights into the causes and outcomes of disease and enhanced disease prediction and prevention.

KEY CONCEPT 2. Mobile applications Mobile apps have the ability to improve efficiency and data collection for planners and the possibility to enhance public participation in local governance. Apps that track running and walking routes are being investigated for where populations move and how parks and other built environment infrastructure may be associated with such movement (12, 53). For example, mobile apps like My Fitness Pal, MapMyRun, MapMyWalk, etc. have been developed to record users’ walking, bicycling routes, recreation habits, and commute mode choices (54). Another mobile app, Lark, tracks workouts via smartphone sensors and also acts as a personal coach and cheerleader that users can communicate and talk with. The app analyses a user’s daily activities, suggests workouts based on those activities, and also sends encouraging texts to motivate the user to exercise and eat healthy.

KEY CONCEPT 3. Wearable technology Wearable technology (also called wearables or wearable devices) are physical objects (e.g., clothing, accessories like wristbands, bracelets, watches, and clip-ons) embedded with electronics, software, sensors, and connectivity to enable objects to exchange data with a manufacturer, operator, and/or other connected devices, without requiring human intervention (55, 56). In a worldwide survey identifying fitness trends for 2017, the American College of Sports Medicine has ranked wearable technology to be the current top fitness trend (57).

KEY CONCEPT 4. Webcams and imagery databases A webcam has been defined as, “a video camera that feeds or streams its images or videos in real time to or through a computer to a computer network” (58). Captured data from webcams is saved by the computer networks creating large imagery databases. Some webcams, for example, those used as online traffic cameras, may be connected to the Web continuously, and can supply a view for anyone who visits its web page over the internet. Webcams and large-scale imagery databases can provide the data necessary for a richer understanding of the interaction between individuals and their environments.

Several apps have been created to support cities and their planning processes. For example, the Placemeter sensor uses computer vision algorithms to create a real-time data layer about places, streets, and neighborhoods (59). Placemeter transforms live video into structured data about pedestrian, bike, and vehicle traffic. Apps like CounterPoint provide refined data by plotting detailed transportation activity by mode, time, duration of the count, weather, etc., and are capable of improving accuracy and quality of data collection (60). Most mobile apps can be downloaded free of cost by users and are supported across a variety of electronic devices and platforms (e.g., Android, Apple, Windows, Blackberry). These apps freely permit large-scale data collection across widespread geographic areas. The availability of information on routes such as distance, speed, elevation, origin, and destination can allow researchers to conduct further detailed investigation on substantially larger samples of physical activity across extensive geographic locations.

Wearable technology is finding a range of innovative ways to improve our health and well-being. By incorporating computer and advanced electronic technologies, wearables have made technology pervasive by interweaving it into daily life, resulting in a movement referred to as Quantified Self (61). A range of smartwatches, fitness bands, and wearable devices are embedded with built-in sensors and GPS that measure individuals’ physical activity as well as the locations where it occurs. Activity trackers like FitBit (62), JawBone (63), and Nike Fuelband (64) are able to count steps and calories burned. In addition, these gadgets may measure heart rate, distance traveled, speed, altitude, calories consumed, floors climbed, skin temperature, and even an individual’s sleep patterns the night before. Wearables can track different types of activities and workouts, such as walking, hiking, bicycling, yoga, and weight lifting, and also have the ability to alert users when they have been sedentary for too long. Some trackers are also able to learn users’ baseline activity level and suggest appropriate personalized goals.

Another type of wearable technology are wearable cameras that provide digital snapshots of everyday life activities by automatically capturing images from a first-person point of view. They include a wide-angle lens that is designed to take photographs passively, at periodic intervals without user intervention, while it is being worn by users. Examples include the Microsoft SenseCam (65, 66), Narrative Clip, and Google Glass (67). Studies have shown the feasibility of using digital life-log images to investigate active and sedentary behaviors.

An example of an imagery database is the archive of many outdoor scenes (AMOS), a Washington University project, which aims to capture and archive time-lapse images from every publicly available, online, outdoor webcam (e.g., traffic cams, campus cams, ski-resort cams) (68). This dataset was developed in 2006 primarily as a basis to research computer vision algorithms for geo-locating and calibrating cameras, and as a demonstration that webcams can be re-purposed as a complement to satellite imaging for large-scale climate measurement (68). Images are digitally captured from each camera every 30 min and archived in a searchable dataset. This large imagery database allows anyone with a computer and Internet access to view at great detail many locations in the world. Many of the captured environments are urban street intersections that show built environment improvements such as crosswalks and bicycle lanes (11). AMOS provides a unique opportunity to annotate built environment changes and associated human behaviors such as walking, bicycling, and the use of public transportation.

## Discussion

Traditional surveys and observation methods were the mainstay of 20th century scientific research and data collection in the social sciences. After decades of dwindling response rates, an exciting new era appears to be dawning with the rise of emerging technologies and big data. These novel technologies using big data or non-traditional sources may be feasible for filling gaps and limitations in traditional data collection. The development of emerging technologies are evidence of computing systems that have left the confines of a desktop and are spilling out onto the sidewalks, streets, and public spaces of the city. Information processing has now become embedded in and distributed throughout the material fabric of everyday urban space.

Policymakers and active living advocates require a baseline understanding of current use of public spaces to inform planning and resource allocation, and ultimately increase the safety and utility of the built environment for all (69, 70). The application of big data and emerging technologies offer promising techniques for evaluating the effects of built environment interventions on active living. By providing data on physical activity as well the environments where the activity occurs, these emerging technologies can offer important contributions to the field of physical activity and built environment research.

### Strengths and Limitations

There are several notable strengths associated with emerging technologies for measuring and evaluating physical activity. Emerging technologies can provide accurate and continuous measurements of how people and vehicles move about public places. These technologies have the potential to develop data sets that are continuous, temporally rich, and contextualized. The ease of use and transferability can significantly enhance external validity of measures and findings. The extensive and objective nature of this data has potential for tracking of physical activity over large geographic areas and across longer time spans, thus enabling longitudinal studies in future research. This presents an opportunity to understand active living in a variety of environments in an easy, cost-effective, and accurate manner that can inform neighborhood design and placemaking efforts.

Despite the above advantages, the use of big data and emerging technologies for health behavior research presents several limitations. The use of emerging technologies is known to vary by socioeconomic status, gender, age, ethnicity, and other demographic factors. Certain populations may be more likely to use emerging technologies than others. For example, low-income ethnic minority populations may have limited access to resources and awareness of technologies like fitness trackers, wearables, and mobile apps to map their physical activity behaviors. Emerging research on big data and technologies has predominantly studied urban settings and additional research is required to examine its implications in rural settings (26, 47).

Another limitation of wearable technologies is their dependency on individuals to upload data, allow access to data, and/or agree to wear multiple devices. Public access to data from mobile apps and webcams may be restricted since users can decline to make their data publicly available (71). Populations who use emerging technologies to monitor their activity and track their movement may be comparatively more health conscious than the general population. Another disadvantage in the use of technologies to track physical activity is the potential to influence behavior among its users. Studies have shown that some users may be motivated to increase physical activity levels, while others may feel discouraged and abandon the tracker (72). Additional limitations include the inability of data and technologies to truly capture individual demographics as well as knowledge and attitudes of users (73). Overall, the adoption of useful technologies can increase the amount and quality of global recorded measurements of physical activity patterns and the potential to effectively design environments that promote active living and healthy behaviors.

## Conclusion

Emerging technologies demonstrate great potential to improve the ongoing, systematic collection, and analysis of public health surveillance data due to real-time monitoring capabilities. As new technologies continue to develop, it is crucial that researchers and practitioners across community health and planning fields collaborate to explore various environments, interventions, and healthy behaviors. Future research should focus on the development of the use of emerging technologies in order to establish image-based baseline data, as well as provide generalizability to the method. Harnessing emerging technologies can bring researchers, practitioners, professionals, policy makers, as well as people to engage in their own data, with significant potential for promotion and evaluation of active living. By employing cutting-edge methods in new settings, rapid and effective evaluation of policies and built environment changes can be conducted, fostering a transdisciplinary understanding across related academic disciplines (e.g., urban design/planning, geography) as well as the advancement of methodologies in computer science and public health. These methods have the ability to greatly improve the generalizability of public health surveillance for active living research.

## Author Contributions

DA is the sole author and is responsible for the content and material discussed in this focused review.

## Conflict of Interest Statement

The author declares that the research was conducted in the absence of any commercial or financial relationships that could be construed as a potential conflict of interest.
